# From microplastics to disinfection by-products: A risky transformation in water treatment

**DOI:** 10.1016/j.eehl.2026.100234

**Published:** 2026-03-12

**Authors:** Yaxuan Liu, Honghong Lyu, Jingchun Tang

**Affiliations:** a MOE Key Laboratory of Pollution Processes and Environmental Criteria/Tianjin Engineering Center of Environmental Diagnosis and Contamination Remediation, College of Environmental Science and Engineering, Nankai University, Tianjin 300350, China; b Tianjin Key Laboratory of Clean Energy and Pollution Control, School of Energy and Environmental Engineering, Hebei University of Technology, Tianjin 300401, China

Microplastics (MPs), including their smaller fraction known as nanoplastics (NPs), are a class of globally emerging contaminants and are ubiquitously present across various water treatment systems [1]. Due to the limited removal efficiency of existing water treatment processes—particularly for NPs—approximately 10%–30% of MPs bypass preliminary treatment and enter the disinfection stage [2]. Upon exposure to disinfectants such as chlorine and ozone, MPs undergo a series of transformations, including surface oxidation, fragmentation, and the release of dissolved organic matter (DOM), which act as non-traditional precursors in the formation of disinfection by-products (DBPs) [3].

Significant knowledge gaps remain regarding the formation mechanisms and risks of MP-derived DBPs (MP-DBPs), which threaten human health through drinking water and aquatic ecosystems via wastewater discharge [4]. Compared to natural organic matter, MP-derived DOM exhibits higher reactivity in DBP formation [5], generating trihalomethanes and haloacetic acids at levels exceeding regulatory limits during chlorination (U.S. EPA regulatory limits: 0.080 and 0.060 mg/L, respectively) [6]. Moreover, plastic additives—such as plasticizers, antioxidants, and flame retardants—can undergo halogenation during disinfection, forming structurally novel DBPs with poorly characterized toxicity [7]. Some evidence suggests these derivatives may possess greater acute and chronic toxicity than the parent MPs, implying that their impacts could be underestimated [8]. These knowledge gaps—especially regarding the systematic identification of novel MP-DBPs, their formation and transformation pathways, and their chronic, mixture, and intergenerational toxicities—impede comprehensive risk assessment and the development of targeted management strategies.

To address this emerging risk, an integrated, interdisciplinary approach is urgently required. Researchers must prioritize closing these knowledge gaps through mechanistic and toxicological studies. Technologists should advance scalable treatment methods—such as advanced oxidation, membrane separation, and adsorption—to limit precursor release at the source. Regulatory agencies, in collaboration with scientific institutions, need to establish evidence-based frameworks to prioritize control measures and support risk governance. Through such concerted efforts, we can advance the understanding of MP-DBPs, refine risk assessment, and implement effective management to safeguard water security and public health in the plastic age.

## CRediT authorship contribution statement

**Yaxuan Liu:** Writing – review & editing, Writing – original draft, Conceptualization. **Honghong Lyu:** Writing – review & editing, Resources. **Jingchun Tang:** Writing – review & editing, Supervision, Funding acquisition.

## Declaration of competing interest

The authors declare that they have no known competing financial interests or personal relationships that could have appeared to influence the work reported in this paper.
